# Do emergency medicine journals promote trial registration and adherence to reporting guidelines? A survey of “Instructions for Authors”

**DOI:** 10.1186/s13049-016-0331-3

**Published:** 2016-11-24

**Authors:** Matthew T. Sims, Nolan M. Henning, C. Cole Wayant, Matt Vassar

**Affiliations:** Oklahoma State University Center for Health Sciences, Tulsa, OK USA

**Keywords:** Reporting guidelines, EQUATOR network, ICMJE, CONSORT, PRISMA, STROBE, STARD, Clinical trial registry, ClinicalTrials.gov, WHO

## Abstract

**Background:**

The aim of this study was to evaluate the current state of two publication practices, reporting guidelines requirements and clinical trial registration requirements, by analyzing the “Instructions for Authors” of emergency medicine journals.

**Methods:**

We performed a web-based data abstraction from the “Instructions for Authors” of the 27 Emergency Medicine journals catalogued in the Expanded Science Citation Index of the 2014 Journal Citation Reports and Google Scholar Metrics h5-index to identify whether each journal required, recommended, or made no mention of the following reporting guidelines: EQUATOR Network, ICMJE, ARRIVE, CARE, CONSORT, STARD, TRIPOD, CHEERS, MOOSE, STROBE, COREQ, SRQR, SQUIRE, PRISMA-P, SPIRIT, PRISMA, and QUOROM. We also extracted whether journals required or recommended trial registration. Authors were blinded to one another’s ratings until completion of the data validation. Cross-tabulations and descriptive statistics were calculated using IBM SPSS 22.

**Results:**

Of the 27 emergency medicine journals, 11 (11/27, 40.7%) did not mention a single guideline within their “Instructions for Authors,” while the remaining 16 (16/27, 59.3%) mentioned one or more guidelines. The QUOROM statement and SRQR were not mentioned by any journals whereas the ICMJE guidelines (18/27, 66.7%) and CONSORT statement (15/27, 55.6%) were mentioned most often. Of the 27 emergency medicine journals, 15 (15/27, 55.6%) did not mention trial or review registration, while the remaining 12 (12/27, 44.4%) at least mentioned one of the two. Trial registration through ClinicalTrials.gov was mentioned by seven (7/27, 25.9%) journals while the WHO registry was mentioned by four (4/27, 14.8%). Twelve (12/27, 44.4%) journals mentioned trial registration through any registry platform.

**Discussion:**

The aim of this study was to evaluate the current state of two publication practices, reporting guidelines requirements and clinical trial registration requirements, by analyzing the “Instructions for Authors” of emergency medicine journals. In this study, there was not a single reporting guideline mentioned in more than half of the journals. This undermines efforts of other journals to improve the completeness and transparency of research reporting.

**Conclusions:**

Reporting guidelines are infrequently required or recommended by emergency medicine journals. Furthermore, few require clinical trial registration. These two mechanisms may limit bias and should be considered for adoption by journal editors in emergency medicine.

**Trial registration:**

UMIN000022486

**Electronic supplementary material:**

The online version of this article (doi:10.1186/s13049-016-0331-3) contains supplementary material, which is available to authorized users.

## Background

Reporting guidelines have been developed for authors to improve the quality of research reporting, encourage transparency, and discourage methodological aspects of study design that contribute to bias [1, 2]. Hirst and Altman call the poor reporting plaguing medical research “unethical, wasteful of scarce resources and even potentially harmful” [3] and suggest that guideline adoption may help mitigate these issues. Evidence suggests that guideline adoption improves the quality of research reporting [4, 5], in part, by minimizing the omission of critical information in methods sections, inadequate reporting of adverse events, or misleading presentations of results [6]. The EQUATOR (Enhancing the QUAlity of Transparency Of health Research) Network is an international initiative devoted to advancement of high quality reporting of health research studies [7]. To date, EQUATOR has catalogued 308 guidelines for all types of study designs [7]. CONSORT guidelines for randomized controlled trials, PRISMA guidelines for systematic reviews, and STROBE guidelines for observational studies are among the most used guidelines found in the EQUATOR library.

Clinical trial registration has also been recognized as a means to limit bias [8, 9]. A large body of evidence suggests, for example, that selective reporting of outcomes is a common problem across many areas of medicine [10]. The International Committee of Medical Journal Editors (ICMJE) require all journals within its network only accept manuscripts for publication if prior trial registration occurred before the first patient was enrolled [11]. The World Health Organization has also released a position statement supporting trial registration [12], and the United States government enacted the FDA Amendments Act requiring such registrations. Therefore, the requirement by journals to register clinical trials coupled with guidance on use of reporting guidelines may lead to improved study design and reporting. Here, we investigate the policies of Emergency Medicine journals concerning guideline adoption and clinical trial registration to understand the extent to which journals are using these mechanisms as a means to improve reporting practices.

## Methods

We conducted a review of journal policies and “Instructions for Authors” concerning guideline adherence and trial registration requirements. This study did not meet the regulatory definition of human subject research as defined in 45 CFR 46.102(d) and (f) of the Department of Health and Human Services’ Code of Federal Regulations and, therefore, was not subject to Institutional Review Board oversight. We applied relevant SAMPL guidelines for reporting descriptive statistics [13]. This study is registered on the University hospital Medical Information Network Clinical Trial Registry (UMIN-CTR, UMIN000022486) and data from this study is publically available on figshare (https://dx.doi.org/10.6084/m9.figshare.3406477.v6).

We conducted a search of two journal indexing databases, the Expanded Science Citation Index of the 2014 Journal Citation Reports [14–23] (Thomson Reuters; New York, NY) accessed on February 17, 2016 and Google Scholar Metrics h5-index [23–27] emergency medicine subcategory (Google Inc; Mountain View, CA) accessed on October 11, 2016. Between the two databases, we selected 27 emergency medicine journals. Additional file 1: Table S1 details journal selection. The second author (NMH) performed web-based searches for each journal and located the “Instructions for Authors” information (performed June 4, 2016 and October 11, 2016). The first (MTS) and last (MV) authors reviewed together each journal’s website to determine the types of articles accepted for publication. In many cases, the descriptions were vague and required further clarification. These authors emailed the Editor-in-Chief of the journals to inquire about the types of study designs considered for publication (systematic reviews/meta-analyses, clinical trials, diagnostic accuracy studies, case reports, epidemiological studies, and animal studies). For non-responding editors, we emailed three times in one week intervals in an attempt to obtain this information as suggested by Dillman to improve response rates [28]. A second round of emails were sent to the Editor-in-Chief of the journals to inquire about an additional set of study designs considered for publication (qualitative research, quality improvement studies, economic evaluations, and study protocols). For non-responding editors, follow up emails were sent in a one-week interval as well to maintain consistency in data obtaining methods.

Prior to the study, all authors met to plan the study design and discuss any anticipated problems. Follow-up meetings were held to pilot test the process and address any issues that arose after evaluating a subset of journal guidelines. Adjustments were made as necessary based on consensus of the authors, and a final extraction manual was created to standardize the process.

The second author (NMH) first catalogued the journal title, impact factor, and geographic location (defined by the primary location of the journal’s editorial office as indexed in the Expanded Science Citation Index) for each journal. We followed the classification of Meerpohl et al. [22] and classified geographic location as either North America (United States and Canada), the United Kingdom, Europe (without the United Kingdom), and other countries. Next, this author used a combination of keyword searches and reviewed the full-text versions of the “Instructions for Authors,” Submission Guidelines, Editorial Policies, or other relevant sections related to the manuscript submission (hereafter referred to as “Instructions for Authors”). The policy statements for the following guidelines were extracted: Consolidated Standards of Reporting Trials (CONSORT), Meta-Analysis of Observational Studies in Epidemiology (MOOSE), Quality of Reporting of Meta-analyses (QUOROM), Preferred Reporting Items for Systematic Reviews and Meta-Analyses (PRISMA), Standards for Reporting Diagnostic Accuracy Studies (STARD), Strengthening the Reporting of Observational Studies in Epidemiology (STROBE), Animal Research: Reporting of In Vivo Experiments (ARRIVE), Case Reports (CARE), Consolidated Health Economic Evaluation Reporting Standards (CHEERS), Standards for Reporting Qualitative Research (SRQR), Standards for Quality Improvement Reporting Excellence (SQUIRE), Standard Protocol Items: Recommendations for Interventional Trials (SPIRIT), Consolidated Criteria for Reporting Qualitative Research (COREQ), Transparent Reporting of a Multivariate Prediction Model for Individual Prognosis or Diagnosis (TRIPOD), Preferred Reporting Items for Systematic Review and Meta-Analysis Protocols (PRISMA-P) and the International Committee of Medical Journal Editors (ICMJE) guidelines. Information regarding clinical trial registration and review registration requirements and acceptable or recommended trials and review registries, when provided, were also extracted. Statements that were extracted in a language other than English were translated using Google Translate (Google Inc; Mountain View, CA).

Following data extraction, the first (MTS) and third (CCW) authors examined each extracted statement and, using a Google form, rated whether the journal required, recommended, or failed to mention each reporting guideline. Statements about trial registration were rated the same way. During the pilot session, it was agreed that words or phrases such as “should,” “prefer,” “encourage,” or “in accordance to the recommendation of” were rated as recommended, while words or phrases such as “must,” “need,” or “manuscripts won’t be considered for publication unless” were regarded as required/compulsory. If the authors (MTS and CCW) were unable to determine whether a journal required or recommended a particular guideline, then the statement was marked as unclear. Each author was blinded to the ratings of the other. MTS and CCW met after completing the rating process to compare scores and resolve any disagreements. Cross-tabulations and descriptive statistics were calculated using IBM SPSS 22 (IBM Corporation; Armonk, NY). If a journal did not publish a particular type of study, then it was not considered when computing proportions. For example, if a journal did not publish preclinical animal studies, then the ARRIVE Guidelines were not relevant to that journal.

## Results

Our sample was comprised of 27 emergency medicine journals. The impact factors of these journals ranged from 0.200 to 4.695 [x̄ = 1.504 (SD = 1.145)]. Editorial offices were located in North America (15/27, 55.6%), Europe (6/27, 22.2%), the United Kingdom (4/27, 14.8%), and other countries (2/27, 7.4%). For each study type, the appropriate guideline was identified (Table 1). Following a review of “Instructions for Authors” and editor-in-chief email inquiries (response rate = 13/27, 48.1%), the following reporting guidelines were removed from computing proportions due to their study type not being accepted by the journal: CARE statement (7/27, 25.9%), STARD (1/27, 3.7%), TRIPOD statement (1/27, 3.7%), MOOSE checklist (1/27, 3.7%), STROBE checklist (1/27, 3.7%), and SRQR (2/27, 7.4%) (Table 2).

**Table 1:** Reporting guidelines by study type

Animal Research	ARRIVE Guidelines
Case Reports	CARE Guidelines
Clinical Trials	CONSORT Statement
Diagnostic Accuracy Studies	STARD
	TRIPOD Statement
Economic Evaluations	CHEERS Statement
Observational Studies in Epidemiology	MOOSE Statement
	STROBE Statement
Qualitative Research	COREQ Checklist
	SRQR
Quality Improvement Studies	SQUIRE Checklist
Study Protocols	PRISMA-P Statement
	SPIRIT Statement
Systematic Reviews & Meta-Analyses	PRISMA Statement
	QUOROM Checklist

**Table 2:**
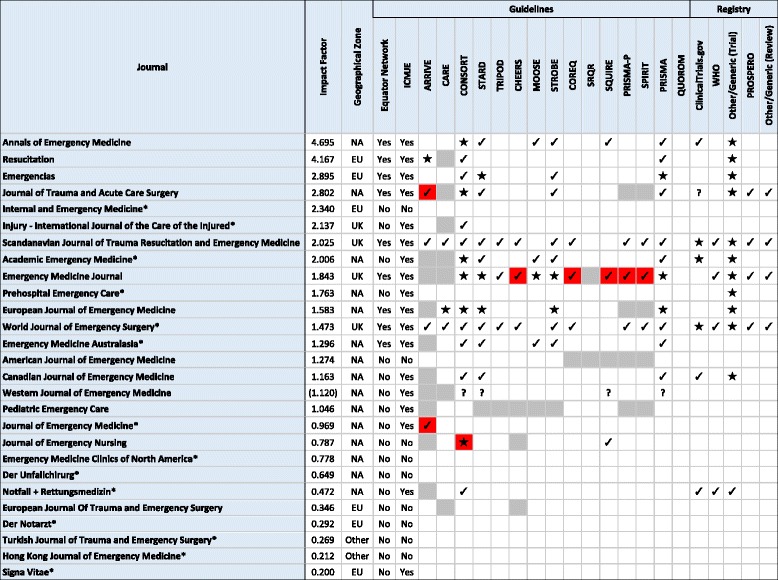
Use of reporting guidelines and study registration by journal

★ = Required/Compulsory, ✔ = Recommended, **?** = Unclear, **Boxes filled with grey** = journal does not accept the study design requiring these guidelines, **Boxes filled with red** = journal does not accept the study design yet the “Instructions for Authors” mention the guideline, * = editor in chief did not respond to email inquiry. **(Impact Factor) =** self-calculated impact factor by the journal. All data was extracted on June 4, 2016 except *Journal of Trauma and Acute Care Surgery*, *Internal and Emergency Medicine*, & *Western Journal of Emergency Medicine* which data extraction occurred on October 27, 2016

### Reporting guidelines

The “Instructions for Authors” of nine (9/27, 33.3%) journals referenced the EQUATOR Network. The authors’ guidelines of 18 (18/27, 66.7%) journals referenced the ICMJE guidelines. Of the 27 emergency medicine journals, 11 (11/27, 40.7%) did not mention a single guideline within their “Instructions for Authors,” while the remaining 16 (16/27, 59.3%) mentioned one or more guidelines.

**Fig. 1:**
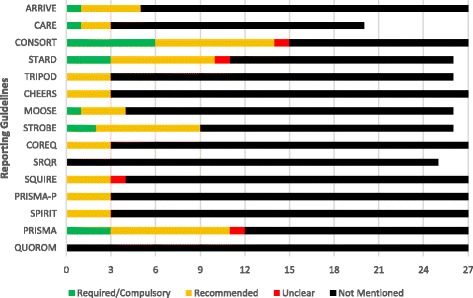
Frequency of reporting guideline adherence across journals. This figure depicts the adherence to each guideline reviewed within this study. Studies that weren’t accepted by journals had their guidelines removed when computing proportions

Guideline usage is presented in Table 2. Across reporting guidelines, the CONSORT statement (15/27, 55.6%) was most frequently required (6/15, 40.0%) and recommended (8/15, 53.3%) by journal in our sample followed by the PRISMA guidelines (12/27, 44.4%) and STARD guidelines (11/27, 40.7%). The QUOROM statement and SRQR were not mentioned by any journals (Fig. 1).

Adherence to each of the following guidelines was mentioned within the “Instructions for Authors” of an emergency medicine journal; however, the editor-in-chief for the respective journal listed the guideline’s study type as unaccepted. The ARRIVE statement was mentioned by four journals: one (1/4, 25.0%) required adherence and three (3/4, 75.0%) recommended adherence. The SQUIRE checklist was mentioned by four journals: three (3/4, 75.0%) recommended adherence and one (1/4, 25.0%) had an “unclear” adherence statement. The CHEERS statement, SPIRIT checklist, COREQ checklist, and PRISMA-P statement were each mentioned by three journals: every journal recommended adherence (Table 2).

### Study registration

**Fig. 2:**
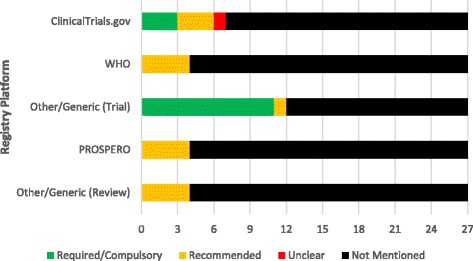
Frequency of registration adherence across journals. This figure depicts the adherence to each type of trial and/or review registriation

Of the 27 emergency medicine journals, 15 (15/27, 55.6%) did not mention trial or review registration, while the remaining 12 (12/27, 44.4%) at least mentioned one of the two. Trial registration through ClinicalTrials.gov was required by three (3/27, 11.1%) journal, recommended by three (3/27, 11.1%) journals, and one (1/27, 3.7%) journal contained an “unclear” adherence statement. Registration through World Health Organization (WHO) was recommend by four (4/27, 14.8%) journals. Eleven (11/27, 40.7%) journals required trial registration through any trial registry and one (3.7%, 1/27) journals recommended registration. Review registration through the PROSPERO platform was recommended by four (4/27, 14.8%) journals. Review registration through any review registry platform was recommended by four (4/27, 14.8%) journals (Fig. 2).

## Discussion

The aim of this study was to evaluate the current state of two publication practices, reporting guidelines requirements and clinical trial registration requirements, by analyzing the “Instructions for Authors” of emergency medicine journals. In this study, there was not a single reporting guideline mentioned in more than half of the journals. This undermines efforts of other journals to improve the completeness and transparency of research reporting. For example, the 2001 CONSORT revision states, “…inadequate reporting borders on unethical practice when biased results receive false credibility” [29]. Furthermore, it could be argued that it is the moral duty of researchers and reviewers to report their findings as clearly as possible to allow readers the ability to accurately judge the validity of the results [29–33]. The scientific process demands transparency and without it, authors are blinding their readers [29, 31, 32]. While blinding participants and researchers is a significant aspect of study quality, blinding readers impairs the scientific inquiry [29, 31, 32].

In this study, we assessed whether journals continued to mention adherence to the QUOROM statement even though it was superseded by the PRISMA statement in 2009 [34]. Our analysis of emergency medicine journals indicated that none of the journals mentioned adherence to the QUOROM statement. Evidence suggests that a few journals continue to refer to the QUOROM statement [18].

In 2006, the EQUATOR Network was developed with the intent of improving the value and reliability of research literature by promoting accurate and transparent reporting [7]. The EQUATOR Network provides access to guidelines including: CONSORT, STROBE, PRISMA, CARE, STARD, ARRIVE, and others. With the support of major editorial groups, guidelines such as CONSORT, STROBE, and PRISMA are recognized by high impact factor journals [30]. Hopewell et al. [35] reported that between 2000 and 2006, journals that adopted these guidelines displayed an improvement in reporting of critical elements. While there has been an improvement in trial quality throughout the research field, evidence suggests that journals adopting the CONSORT guidelines have been improving at a faster pace than journals that did not [36].

While reporting guidelines are invaluable for achieving accurate, complete, and transparent reporting, trial registration is a major contributor as well. In a recent British Medical Journal (BMJ) publication, medical journal editors expressed their firm belief that required trial registration is the single most valuable tool in ensuring unbiased reporting [37]. In 2007, clinical trial registration became a requirement of investigators in accordance with United States (US) law [Public Law 110–85, Title VIII, also called the FDA Amendments Act (FDAAA) of 2007, Section 801] [38, 39]. However, a significant number of researchers and journal editors are reluctant to completely endorse trial registration [40–42]. and enforce this existing legislation [43, 44]. In an attempt to reduce publication bias, the ICMJE and CONSORT statement require trial registration in order to be considered for publication [45, 46]. Regardless of these requirements and US legislation, one in four published studies is not registered allowing for potential selective reporting bias [47]. Trial registration remains a problem. Recent studies have indicated that many publish RCTs are unregistered or prospectively registered, and rates of trial registration vary by clinical specialty [48–55]. These practices might be improved if journals would develop and adhere to policies requiring prospective registration prior to submission. Our study found that most emergency medicine journals do not have such policies.

In some fields of medical research, such as rehabilitation and disability, the journals have formed a collaboration to enhance research reporting standards [56]. As of 2014, 28 major rehabilitation and disability journals have formed a group to require adherence to reporting guidelines to improve the quality of research reporting not only within their journal, but their field of medicine and research as a whole. This movement has made it difficult for authors receiving unfavorable reviews due to inadequate guideline adherence to pick and choose journals that may present more lax reporting guideline adherence. It would be reasonable for emergency medicine journal editors to consider forming such a collaboration, especially if evidence suggests that such collaborations improve the quality of research reporting.

### Study limitations

In our study, we attempted to contact editors to obtain information when the “Instructions for Authors” were unclear, some did not respond to our inquiries, and we were not able to verify or clarify whether these journals published certain types of study designs.

### Future research

In future studies, researchers could evaluate the practical aspects of using guidelines from both the authors’ and editors’ perspectives. Here, we discuss the use of these reporting guidelines as a means to mitigate bias and provide more complete reporting of study information. The practical aspects of guideline reporting and monitoring, however, are not well understood, and it is conceivable that active monitoring of guideline adherence is not feasible given limited resources. Another interesting line for future research would be to investigate whether guideline adherence improves quality within other study designs such as systematic reviews. Researchers could investigate differences in quality between journals that require guideline adherence, recommend guideline adherence, and omit information about reporting guidelines.

## Conclusion

In conclusion, reporting guidelines are infrequently required or recommended by emergency medicine journals. Furthermore, few require clinical trial registration. These two mechanisms may limit bias and should be considered for adoption by journal editors in emergency medicine. Emergency medicine journals might recommend, rather than mandate, these mechanisms as first steps toward adoption. Additional research is needed to determine the effectiveness of these mechanisms.

## Additional file

Additional file 1: Table S1. Journal selection, with exclusions. (DOCX 13 kb)

## Abbreviations

ARRIVE: Animal Research: Reporting of In Vivo Experiments
BMJ: British Medical Journal
CARE: CAse REports
CHEERS: Consolidated Health Economic Evaluation Reporting Standards
CONSORT: Consolidated Standards of Reporting Trials
COREQ: Consolidated Criteria for Reporting Qualitative Research
EQUATOR: Enhancing the QUAlity of Transparency Of health Research
ICMJE: International Committee of Medical Journal Editors
MOOSE: Meta-Analysis of Observational Studies in Epidemiology
PRISMA: Preferred Reporting Items for Systematic Reviews and Meta-Analyses
PRISMA-P: Preferred Reporting Items for Systematic Review and Meta-Analysis Protocols
QUOROM: Quality of Reporting of Meta-analyses
SPIRIT: Standard Protocol Items: Recommendations for Interventional Trials
SQUIRE: Standards for Quality Improvement Reporting Excellence
SRQR: Standards for Reporting Qualitative Research
STARD: Standards for Reporting Diagnostic Accuracy Studies
STROBE: Strengthening the Reporting of Observational Studies in Epidemiology
TRIPOD: Transparent Reporting of a Multivariate Prediction Model for Individual Prognosis or Diagnosis
US: United States
