# Supplementary material for: Do emergency medicine journals promote trial registration and adherence to reporting guidelines? A survey of “Instructions for Authors”
Source: Scand J Trauma Resusc Emerg Med. 2016 Nov 24;24:137. doi: 10.1186/s13049-016-0331-3 (PMC5121955; doi:10.1186/s13049-016-0331-3)
Supplement: Additional file 1: Table S1. — Journal selection, with exclusions. (DOCX 13 kb) [file 13049_2016_331_MOESM1_ESM.docx]

**Additional file 1: Table S1. Journal selection, with exclusions**

| **Thomson Reuters Expanded Science Citation Index of the 2014 Journal Citation Reports** | **Google Scholar Metrics h5-Index Subcategory Emergency Medicine** |
| --- | --- |
| **Included Journals (n = 24)** | **Included Journals (n = 3)** |
| Academic Emergency Medicine | Internal and Emergency Medicine |
| American Journal of Emergency Medicine | Journal of Trauma and Acute Care Surgery |
| Annals of Emergency Medicine | Western Journal of Emergency Medicine |
| Canadian Journal of Emergency Medicine |  |
| Der Notarzt | **Duplicate Journals (n = 11)** |
| Der Unfallchirurg | American Journal of Emergency Medicine |
| Emergencias | Annals of Emergency Medicine |
| Emergency Medicine Australasia | Emergency Medicine Journal |
| Emergency Medicine Clinics of North America | European Journal of Emergency Medicine |
| Emergency Medicine Journal | Journal of Emergency Medicine |
| European Journal of Emergency Medicine | Journal of Emergency Nursing |
| European Journal Of Trauma and Emergency Surgery | Pediatric Emergency Care |
| Hong Kong Journal of Emergency Medicine | Prehospital Emergency Care |
| Injury - International Journal of the Care of the Injured | Resuscitation |
| Journal of Emergency Medicine | Scandinavian Journal of Trauma, Resuscitation and Emergency Medicine |
| Journal of Emergency Nursing | World Journal of Emergency Surgery |
| Notfall + Rettungsmedizin |  |
| Pediatric Emergency Care | **Excluded Journals (n = 5)** |
| Prehospital Emergency Care | Clinical Toxicology |
| Resuscitation | Critical Care Clinics |
| Scandinavian Journal of Trauma Resuscitation and Emergency Medicine | Current Opinion in Critical Care |
| Signa Vitae | Disaster Medicine and Public Health Preparedness |
| Turkish Journal of Trauma and Emergency Surgery | Journal of Medical Toxicology |
| World Journal of Emergency Surgery |  |
